# EFFNet-CA: An Efficient Driver Distraction Detection Based on Multiscale Features Extractions and Channel Attention Mechanism

**DOI:** 10.3390/s23083835

**Published:** 2023-04-08

**Authors:** Taimoor Khan, Gyuho Choi, Sokjoon Lee

**Affiliations:** 1 Department of Computer Engineering, Gachon University, Seongnam-si 13120, Republic of Korea; 2 Department of Artificial Intelligence Engineering, Chosun University, Gwangju 61452, Republic of Korea; ghchoi@chosun.ac.kr; 3 Department of Smart Security, Gachon University, Seongnam-si 13120, Republic of Korea

**Keywords:** convolutional neural network, driver distraction detection, driver behavior ANALYSIS, EfficientNetB0, channel attention mechanism

## Abstract

Driver distraction is considered a main cause of road accidents, every year, thousands of people obtain serious injuries, and most of them lose their lives. In addition, a continuous increase can be found in road accidents due to driver’s distractions, such as talking, drinking, and using electronic devices, among others. Similarly, several researchers have developed different traditional deep learning techniques for the efficient detection of driver activity. However, the current studies need further improvement due to the higher number of false predictions in real time. To cope with these issues, it is significant to develop an effective technique which detects driver’s behavior in real time to prevent human lives and their property from being damaged. In this work, we develop a convolutional neural network (CNN)-based technique with the integration of a channel attention (CA) mechanism for efficient and effective detection of driver behavior. Moreover, we compared the proposed model with solo and integration flavors of various backbone models and CA such as VGG16, VGG16+CA, ResNet50, ResNet50+CA, Xception, Xception+CA, InceptionV3, InceptionV3+CA, and EfficientNetB0. Additionally, the proposed model obtained optimal performance in terms of evaluation metrics, for instance, accuracy, precision, recall, and F1-score using two well-known datasets such as AUC Distracted Driver (AUCD2) and State Farm Distracted Driver Detection (SFD3). The proposed model achieved 99.58% result in terms of accuracy using SFD3 while 98.97% accuracy on AUCD2 datasets.

## 1. Introduction

In the past few decades, the rapid increase in road accidents due to the lack of driver attentiveness, has gained researchers’ attention [1]. For instance, in 2016, the World Health Organization (WHO) reported “1.4 million humans lost their lives due to road accidents globally”. In addition, road accident is the eightieth major cause of death [1]. A study by the government of India in 2017, reported that approximately half a million road accidents occurred in India, in which several people lost their lives and many of them obtained serious injuries [2]. In another article reported in 2018 by the Ministry of Road Transport and Highway (MRTH), almost half a million road accidents have been recorded in different states in India, in which roughly 0.15 million people lost lives and almost 0.48 million people obtained serious injuries [3]. Similarly, the report of National Highway Traffic Safety Administration (NHTSA) in the USA concluded that around 64.4% of people lose life due to diversion of attention from driving [3]. Moreover, their report also declared that 94% of car accidents are caused by driver’s inactiveness [3], while a large number of road accidents are due to the usage of electronic devices such as Bluetooth devices, mobile phones, and so on.

Prior studies have demonstrated that drivers’ attention is changed by engaging in other activities when they are driving, which can lead to road accidents. These activities include engaging with electronic devices while driving such as calling, talking, texting, and so on. Researchers are thus motivated to find out the easiest way to reduce the number of road accidents. Therefore, several researchers have presented different computer vision-based methods to alert the driver in case of engaging in other activities while driving. These methods are broadly categories into two major fields such as traditional Machine Learning (ML) and Deep Learning (DL)-based methods [4]. For instance Vural, et al. [5], used a traditional ML approach such as Adaboost and multinomial ridge regression to determine the drivers’ drowsiness based on the 30 facial actions from the Facial Action Coding system. In addition, their resultant technique obtained 90% accuracy across subjects based on two datasets such as, Cohn–Kaneda DFAT-504 and spontaneous expressions dataset. As a follow-up research Babaeian et al. [6], proposed a method by the use of advanced logistic regression using a ML algorithm that can detect driver’s drowsiness based on computing heart rate. Chen et al. [7], used AdaBoost algorithms to fabricate a driving behavior classification model to analyze the behavior of a driver and analyze whether it is safe. In another article, Kumar et al. [8] proposed a method of real-time driver’s drowsiness detection system. The researchers recorded a video through a webcam (Sony CMU-BR300) and detected the driver’s faces using image processing techniques. The researchers used a Support Vector Machine (SVM)-based classification. However, the limited performance, high false alarm rate, and time complexity of traditional ML models are the major factors of failure. Furthermore, in the traditional ML-based models, the handcrafted features extraction and classification are very tedious, error prone, and time-consuming processes. These factors motivated the researchers to explore the DL-based model for driver distraction detection.

For instance, Hssayeni, et al. [9], proposed deep learning models for the detection of drivers’ attentions, although their resultant works require more improvement in terms of accuracy. Kapoor, et al. [10], proposed a light-weight pretrained technique with some fine-tuning strategies for real-time detection of driver distraction. However, their approach generated a false alarm rate due to the rapid movements of the body based on low performance. A DL-based model for drowsiness detection is presented in [11], to determine the driver attentiveness based on facial landmark key point detection. The researchers used the NTHU-DDD dataset and achieved 80% in terms of accuracy. However, the accuracy of their proposed method needs further improvement. 

Driver distraction detection is a problem to be solved, the aforementioned techniques based on traditional ML and DL models are time-consuming and required further enhancement in terms of accuracy and time complexity. In addition, such techniques generate false alarms due to the low performance. Moreover, it is a challenging task to detect driver behavior to overcome road accidents. To deal with the problem in a satisfactory way, we proposed an EfficientNetB0 with CA for the real-time efficient detection of driver distraction. The major contributions of the proposed work are as follows:

- Inspired by the transfer learning technique, we trained different types of pretrained models without dense layers and applied CA mechanism for obtaining optimal performance. In addition, we compared the performance of our proposed model with other architectures including VGG16, VGG16+CA, ResNet50, ResNet50+CA, Xception, Xception+CA, InceptionV3, InceptionV3+CA, and EfficientNetB0.
- The results of a detailed ablation study showed that the EfficientNetB0 with channel attention (CA) achieved the highest performance compared with all other methods. Based on these findings, we selected EfficientNetB0 with CA as the model of choice for driver distraction detection. In addition to its superior performance, the proposed model is also lightweight, enabling fast processing times compared with other architectures. The faster processing time of the EfficientNetB0 with CA mechanism can reduce the risk of accidents and improve the overall safety of drivers and passengers. Furthermore, the lightweight and fast processing nature of the proposed model makes it highly applicable for real-world scenarios that require real-time detection, such as medical diagnosis, video surveillance, and robotics.
- We evaluated the performance of the proposed model on the SFD3 and AUCD2 datasets. Our results showed that the proposed model achieved higher accuracy and faster processing times compared with other baselines. This highlights the potential of the proposed model as a more efficient and effective solution for driver distraction detection in real-world scenarios.

The rest of the article is formatted as follow: in Section 2 we highlight related works with previous literatures and their approaches, Section 3 presents the methodology of our work, discussion and result are available in Section 4, and finally, in Section 5 we provide the conclusion and future work.

## 2. Related Work

Drivers’ distraction is a major cause of accidents that affects human lives and their resources. To cope with these issues, several researchers have proposed different techniques to notify the driver of their distraction based on alarm or messages using a Traditional Deep Learning (TDF) approach. For instance, Alzubia et al. [12], presented a CNN-based method which alerts the drivers by their distraction while driving. In this study, the researcher utilized an ensemble technique to detect driver distraction using their custom dataset. Their method is not only limited to determining drivers’ distractions but also can work in real time using resource constraint edge devices. However, their technique needs further improvement in evaluation matrices. As a follow-up study, Leekha et al. [13] proposed a CNN method and trained the existing method on two publicly available datasets, such as the State Farm Distracted Driver Detection (SFD3) and the AUC Distracted Driver dataset (AUCD2), additionally their proposed method achieved 98.48% and 95.64% performance, respectively. Despite that, their technique is time-consuming as they trained the complex model on datasets. In another research, Varaich et al. [14] used two competing DCNN architectures named InceptionV3, and Xception. In addition, the authors compared the results of both architectures and applied them to recognize ten unique actions of the drivers in the SFD3 dataset. The resultant technique was complicated compared with state-of-the-art techniques. The next method, devised by Jamsheed et al. [3], is a technique for alerting distracted drivers and reducing the ratio of the road accidents based on deep learning. Their technique consists of three models, namely, vanilla CNN, vanilla CNN based on data augmentation, and CNN with transfer learning. Differently, false classification of distraction can happen based on performance. Similarly, Moslemi et al. [1] derived a benefit from temporal information by using a 3D CNN and optical flow to improve the driver monitoring system. Their resultant model achieved 90% performance based on the Kinetics and the SFD3 datasets, but their method is computationally inefficient, in addition, their technique requires further improvement. The next article proposed by Qin et al. [15], introduced a new D-HCNN model based on a declining filter size with only 0.76M parameters, a much smaller number of parameters compared with SOTA based on two available datasets such as AUCD2 and SFD3, through which their model obtained 95.59% and 99.87% performance in terms of accuracy, respectively. 

Another study, presented by Dua et al. [16], was focused on enhancing the performance of four deep learning models: AlexNet, VGG Face, Flow ImageNet, and ResNet. The models detect four types of different features such as hand gestures, facial expressions, behavioral features, and head movements. The authors used NTH Drowsy Driver Detection (NTHU-DDD) video dataset in this article. They passed the RGB videos as input and the goal of that input is detecting the driver drowsiness. Their resultant model achieved 85% accuracy; However, the resultant model is limited in drivers’ behavior classes. 

Alotaibi et al. [17] used a TDF approach and tried to enhance the performance of the proposed model. Moreover, their research is focused on the three popular pretrained CNNs architectures, such as Inception, ResNet, and Hierarchical Multiscale Recurrent Neural Network [18]. Based on Inception, ResNet, and Hierarchical Multiscale Recurrent Neural Network, they obtained promising performance. Additionally, Dhakate et al. [2] implemented four pretrained DL architectures, i.e., VGG16, ResNet50, Xception, and InceptionV3 for the efficient classification of drivers’ distraction, whereas their proposed architecture obtained 97% performance using well-known datasets SFD3 and AUCD2. However, their experiments were performed based on computationally large models such as, VGG16, ResNet50, and so on. The next approach devised by Jabbara et al. [11] proposed a real-time drowsiness detection technique based on Deep Neural Network (DNN). The researchers designed a method using facial landmark key points detection to show whether the driver is active or not. Their work is based on the (NTHU-DDD) dataset and their proposed model obtained 80% accuracy; however, their proposed method requires a proper setup for real-time detection to save the driver privacy.

The approach presented by research Hssayeni et al. [9] utilized a computer vision and ML technique to detect drivers’ behavior based on a dashboard camera. Their experimental results depended on three transfer learning architectures, such as AlexNet, VGG16, and ResNet50 and their proposed model obtained 85% accuracy. However, their proposed architecture creates false detection due to the rapid movement of a body and low accuracy. The other research introduced by Streiffer et al. [19] proposed a convolutional and recurrent neural network that can analyze driving image and IMU sensor data to detect up to six classes of driving behaviors with high performance.

In another study, Valeriano et al. [20] compared different deep learning methods for the classification of driver behavior. However, their proposed method achieved high accuracy of 96.6% based on three rounds of 5-fold cross validation; however, their proposed model needs to deploy edge devices. Masood et al. [21] proposed a CNN-based model that not only detects distraction but also analyzes the images that are captured inside of the vehicle. In addition, their proposed method achieved 99% accuracy using the SFD3 dataset. Furthermore, the VGG16 and VGG19 methods were utilized for the identification of driver distraction in this article. However, their experiments are computationally expensive based on large models. In another approach, Majdi et al. [22] presented an automated supervised learning method called DriveNet for driver distraction detection based on two other popular machine-learning approaches: an Recurrent Neural Network (RNN) and Multi-Layer Perceptron (MLP). Moreover, their presented method reached 95% accuracy, but their experimental setup is complex.

Wöllmer et al. [23] proposed a Long Short-Term Memory (LSTM) technique that figures out real-time distractions of drivers and their resultant technique achieved 96.6% in terms of accuracy; however, the privacy of the driver is a critical issue in real-time distractions. Xing et al. [24] presented a driver behavior recognition system based on DCNN based on a low-cost camera (use for image acquisition). Their work related to three different pretrained CNN architectures, for instance, AlexNet, GoogLeNet, and ResNet50, and their CNN-based models obtained 81.6%, 78.6%, and 74.9%, respectively. These models are also trained for binary classification problems whether the driver is distracted or not. The binary classification rate achieved 91.4% accuracy. The summary of the literature is tabulated in Table 1; however, their models need further enhancement for multiclass classification.

Ye et al. [6] implemented a pretrained Xception network as a backbone for features extraction and incorporated channel attention for selection of more optimal features for detecting driver distraction behavior. Their proposed network (SE-Xception) obtained 92.60% performance in terms of accuracy. Another article presented by Liu et al. [7], utilized channel expansion and attention mechanism to improve YOLOv7 (namely CEAM-YOLOv7) for driver distraction detection using an in-vehicle camera. Additionally, their proposed architecture achieved promising performance among SOTA techniques. As a follow-up research, Zhang et al. [8] introduced a novel attention mechanism-based architecture for driver distraction behavior detection in real time. In this paper, the authors evaluated their proposed method using two datasets such as publicly available dataset and their custom dataset. Lin et al. [9] proposed a novel lightweight architecture known as LWANet. In other words, to decrease the computation cost and number of parameters that can be trained, the classic VGG16 architecture is optimized by reducing its trainable parameters by 98.16% through replacing standard convolution layers with depth-wise separable convolutions. Moreover, the proposed LWANet achieved 99.37% accuracy on SFD3 dataset and 98.45% accuracy using AUC dataset. Another study presented by Wei et al. [10] presented a technique named ENet-CBAM which is based on EfficientNet and Convolutional Block Attention Module for effective detection of driver distraction. Overall, their proposed ENet-CBAM is capable of detecting effectively driver distraction in a real-time scene with few parameters. Similarly, Hu et al. [11] proposed a deep learning-based technique to learn dominant features from the input data. In addition, their proposed technique is improved by two aspects: firstly, use of a multi-scale convolutional block with various kernel sizes to generate a hierarchical feature vector. They also adopted a maximum selection unit that concatenates multi-scale information in an adaptive manner. Secondly, the researchers added an attention mechanism to learn pixel and channel saliency between convolutional features. Furthermore, their experimental results demonstrated that the proposed technique (MSA-CNN) achieved higher performance for driver distraction behavior recognition.

As evident from the literature, numerous researchers have proposed several methods for driver distraction detection. It is worth noting that these techniques suffer from substantial shortcomings including limited performance and required huge computational hardware. In addition, such techniques generate false alarms due to the rapid movement of the body owing to their low performance. Furthermore, the selection of a suitable DL model to deploy over a resource constraint device in real time is a challenging task. To cope with this, in the upcoming section, we briefly explained the proposed model that can be easily deployed over resource constraint devices and can improve the performance over SOTA methods.

## 3. Proposed Method

**Figure 1:**
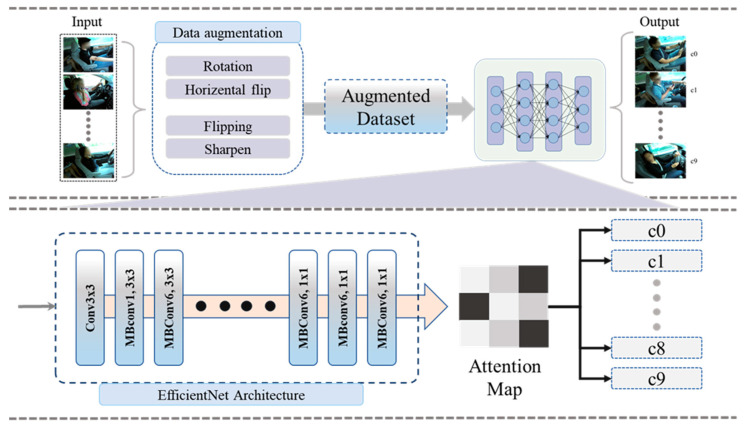
Proposed DL model-based framework for real-time detection of driver’s distraction.

We provided a brief discussion about the proposed model to solve the aforementioned problems in a satisfactory way. The proposed model is composed of two main steps such as (1) preprocessing, to prepare data for training and testing, and (2) training the traditional DL model for accurate driver distraction detection. Furthermore, we fine-tuned a pretrained DL model to enhance the driver distraction detection performance and minimize the false alarm rate. In the proposed work, we employed a CA module with several DL models and validated their performance against SOTA over the benchmark datasets. The proposed framework employing the CA module as is presented in Figure 1. The following subsequent sections explain the details of step 1 and step 2.

### 3.1. Preprocessing

Data preprocessing has a vital role in the ML and DL models and is considered as a fuel for their training [25,26,27,28]. Data preprocessing is a technique for cleaning and organizing unusual data to make them well-known information. In simple words, data preprocessing is a task of data mining that prepares the raw data into an understandable form for model training [29]. Furthermore, the useful and error-free data provide optimal results at the time of evaluation. Additionally, there are several techniques of preprocessing, for instance, augmentation, enhancement, data transformation, and data reduction, among others.

#### Data Augmentation

Data augmentation is a technique that prevents ML and DL models from acquiring irrelevant information. In addition, ML and DL models require a huge amount of data (which are not available easily) for predicting accurate results. In some cases, the available datasets are expanded artificially by applying augmentation techniques [30]. After applying the augmentation technique, the network learns the same object located in the picture with a different view, which enhances the performance of the model [31]. 

Furthermore, there are different steps available in geometric augmentation, for instance, resizing, cropping, rotating, flipping, scaling, and so on. These transformations expand the available dataset and bring the network toward optimal results.

Resizing plays a major role to train any ML or DL models. In addition, our traditional DL models train very quickly and accurately on small images. Moreover, all the DL models need the images to be the same size. The mathematical formulation of resizing is provided below:

(1) $$\left(w_{new},h_{new}\right)=\frac{M}{\max\left(w,h\right)}\left(w,h\right)$$

Normalization is a scaling technique of translating the low and high intensity pixel into the range of 0 and 1 called Min-Max scaling. It mainly keeps the numerical data in a specific range without changing its shape.

(2) $$X_{normalized}=\frac{x-mean\left(x\right)}{x_{max}-x_{min}}$$

Horizontal flip means “flip” or “mirror” look. Horizontal flipping means transforming all the layers of images horizontally, from left to right or right to left. It only changes the position of the pixel on *x*-axis without losing any information.

(3) $$Horizontal\;\left(f\left(x\right)\right)=x^{2}$$

Rotation is a method which is applicable to rotate the object around the center, which simply means, rotate the images in a clockwise or counterclockwise direction. However, we rotated the images 10° in a clockwise position to generate new images. 

Image enhancement is a method used to process the image adjustment, so the resultant image looks more suitable. This method is implemented on input images to avoid noise from an image. The equation is formulated below:

(4) $$g\left(x,y\right)=\left\{\begin{array}{c} a_{1}f\left(x,y\right)f\left(x,y\right)<r_{1}\\ \begin{array}{c} a_{2}\left(f\left(x,y\right)-r_{1}\right)+s_{1},r_{1}\geq f\left(x,y\right)<r_{2}\\ a_{3}\left(f\left(x,y\right)-r_{2}\right)+s_{2},f\left(x,y\right)<r_{2} \end{array} \end{array}\right\}$$

In the above equation, $g\left(x,y\right)$ is the output of the image, while $f\left(x,y\right)$ is the input pixel data; where $a_{1},a_{2}\;\mathrm{and}\;a_{3}$ are scaling factors for many grayscale areas and $s_{1}$,$s_{2},\;r_{1}\;\mathrm{and}\;r_{2}$ are the adjustable parameters.

### 3.2. The Proposed Model

We utilized EfficientNetB0 as a backbone architecture followed by a CA module to increase the performance over the state-of-the-art models. The EfficientNet was proposed latterly as a series of eight networks named, such as, B1, B2, B3, up to B7. The top network version B7 on the ImageNet dataset revealed state-of-the-art results in terms of accuracy by achieving 84.4% top-1 accuracy while using 66 million parameters. In addition, $Swishf\left(x\right)=x.sigmoid\;\left(\beta .x\right)=\frac{x}{1+e^{\beta x}}$ is an activation function introduced with the version of EfficientNet architecture [32]. Moreover, the Swish Activation (SA) function has better performance than the ReLU activation. It obtains better performance on deeper networks throughout of challenging datasets [33].

EfficientNet was introduced by the researcher of Google Tan et al., [34], which is based on the inverted bottleneck residual block (MBConv), which was originally proposed with MobileNetV2. The major goal of the EfficientNet block is to enlarge the channels and then squeeze them; this technique diminishes the number of channels for the upcoming layers [28]. Moreover, this network also brings down the computational weight; hence, it works in in-depth separable convolutions.

**Figure 2:**
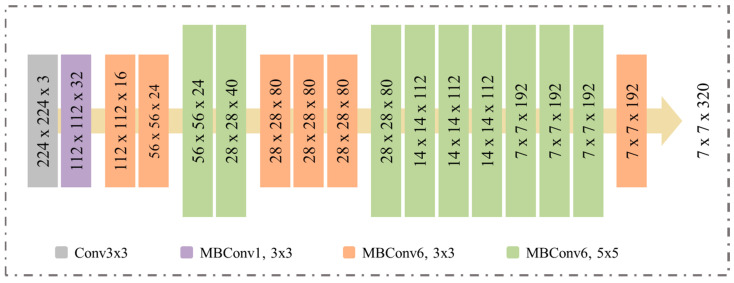
EfficientNetB0 architecture.

Furthermore, we used EfficientNetB0 as a proposed model, which focuses on detecting the driver’s distraction in the early stage based on the optimal performance. On the other hand, EfficientNetB0 is a lightweight architecture where it can easily deploy on edge devices. This model works in block-wise separable convolution neural networks, moreover, it has 237 layers. The proposed model is capable of scaling up or down and it exhibited enormous performance compared with previous state-of-the-art ConvNets [35] on CIFAR-100. The architecture of the proposed model is presented in Figure 2. In the implementation, we used EfficientNetB0 without classification layers, where the features vectors are 7 × 7 with 1280 number of channels (F) and integrated CA mechanism for further strengthening of model performance as is discussed in Section 3.3.

### 3.3. Channel Attention Mechanism

**Figure 3:**
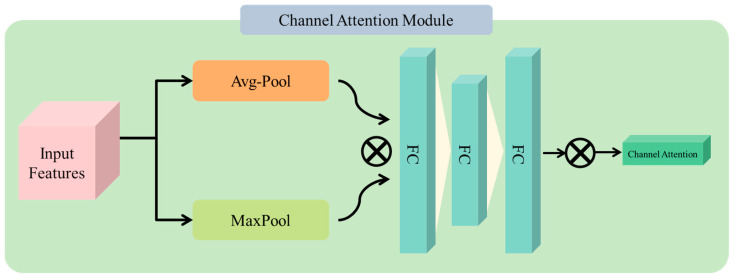
Representation of channel attention mechanism.

To optimally select the features in images that contribute to achieving the targeted task effectively, our experiments were performed by using the CA module between two basic layers to acquire the features. In this article, the CA technique contains global average pooling layer, max-pooling layers, three fully connected layers, and a multiplication operation [36]. However, the main objective of channel attention is to show the relationship between each channel of the feature map and to acquire a 1-D weight $W_{c}\in R^{C\times 1\times 1}$ and then multiply it to a specific channel. For that reason, it can provide more attention to the important information of images in the target task. To learn optimal weights, we used two parallel connections of pooling operation after (F), which is average pooling and max pooling to make two descriptors for each channel. Then we concatenated the output of both channels and fed into shared multilayer perceptron with 3 fully connected layers to create more effective feature vectors. Lastly, we obtained CA by using the SoftMax function as mentioned in Figure 3. The formula is presented as follows:

(5) $$W_{c}\left(F\right)=Softmax\left(MLP\left(AvgPool\left(F\right)\right)+MLP\left(MaxPool\left(F\right)\right)\right)$$

## 4. Results and Discussions

In this section, the results are conducted on two benchmark datasets and the performance of the proposed model is evaluated. First, we provided a detailed explanation about the experimental setup, followed by performance parameters, datasets, and finally presented the results of both datasets in terms of quantitative and qualitative analysis.

### 4.1. Experimental Setup

Our experimental results were conducted in TensorFlow 2.3.0 with Nvidia CUDA support. All the experiments were performed on Ubuntu 20.04.3 LTS operating system, equipped with a Core i7-9700KF CPU, 62 GB Memory, and NVIDIA Corporation TU104 [GeForce RTX 2070 Super GPU] with 8 GB of VRAM.

### 4.2. Performance Parameters

Many frozen CNNs with CA mechanism were used in this study. All the models obtained optimal performance based on a variety of metrics such as testing accuracy, testing loss, F1-score, precision, and recall. True positive (TP), True Negative (TN), False Positive (FP), and False Negative (FN) are the confusion matrix instances, through which we determined the performance of a specific network. Accuracy is a confusion matrix term that indicates the performance of the model for all classes. In simple words, it can measure the number of accurate samples to the total number of samples. The recall is also called sensitivity or True Positive Rate (TPR). This instance evaluates the model to detect driver’s distraction in positive image samples. The specificity of a confusion matrix is determined by the ability to correctly classify negative samples in all true negative cases. Confusion matrix can manage the model that keeps away the model from misidentifying the driver’s distraction. F1-score manages the stability between recall and precision. These matrices are briefly explained in [37,38,39] and the mathematical formulation of these matrices are provided below:

(6) $$Acc\left(k\right)=\frac{TP\left(k\right)+TN\left(k\right)}{TP\left(k\right)+FP\left(k\right)+TN\left(k\right)+FN\left(k\right)}$$

(7) $$Sensitivity\left(k\right)=\frac{TP\left(k\right)}{TP\left(k\right)+FN\left(k\right)}$$

(8) $$F1-Score\left(k\right)=2\left(\frac{precision*recall}{precision+recall}\right)$$

(9) $$Prec\left(k\right)=\frac{TP\left(k\right)}{TP\left(k\right)+FP\left(k\right)}$$

### 4.3. Dataset Description

In this manuscript, the experiments of driver distraction detection were conducted on the two well-known benchmark datasets: the State Farm Distracted Driver Detection [40] (SFD3), which is publicly available; and the AUC Distracted Driver [40] dataset is a private dataset. 

#### 4.3.1. State Farm Distracted Driver Detection (SFD3) Dataset

**Figure 4:**
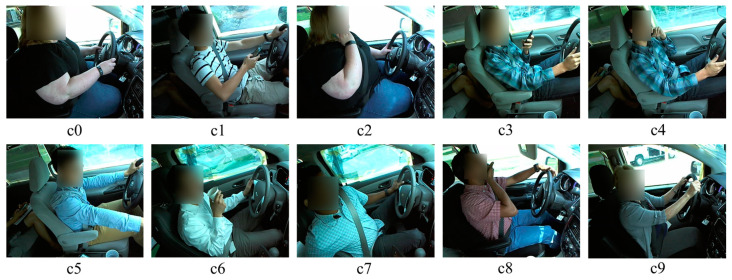
Visual representation of SFD3 dataset where the details of c0~c9 are given in Table 2.

The State Farm Insurance (SFI) company published a challenging dataset of distracted drivers, which is publicly available on the Kaggle competition. The SFD3 contains around 102,150 images of different driver behaviors that are separated into 10 categories as provided in Figure 4, which are labeled as in Table 2. 

#### 4.3.2. AUC Distracted Driver (AUCD2) Dataset

**Figure 5:**
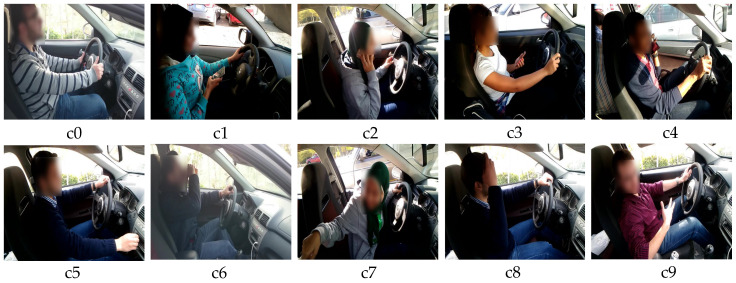
Visual representation of AUCD2 dataset where the details of c0~c9 are given in Table 3.

The AUCD2 is a challenging dataset that was created by Abouelnaga et al. [40], there are thirty-one drivers from different nations, who participated in this dataset. The dataset contains 11,678 images of the different drivers with different postures as tabulated in Table 3; moreover, the images are separated into 10 different folders, where Figure 5 is the visual representation of the AUCD2 dataset. We split both datasets into three sub-sets such as training, testing, and validation. In the training set we have a total of 60% of data, testing 20% of data, and validation 20% of data.

### 4.4. Results Evaluation Using SFD3 Dataset

**Figure 6:**
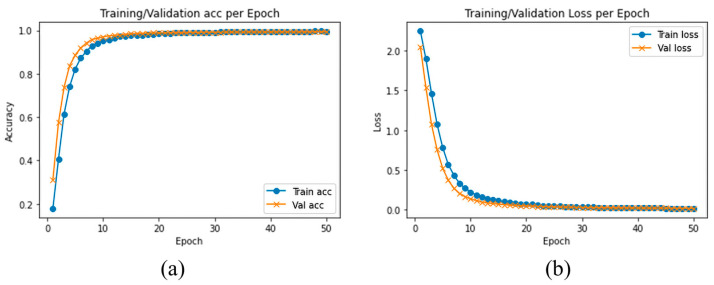
(**a**) Training accuracy and validation accuracy, (**b**) training loss and validation loss using SFD3 dataset.

**Figure 7:**
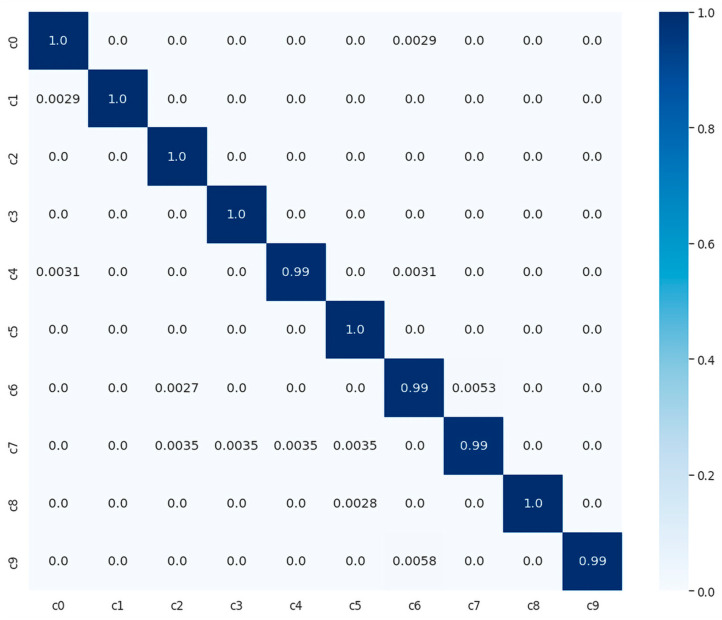
Confusion matrix of proposed model with normalized prediction between zero and one, using SRD3 dataset.

We evaluated and compared the performance of different pretrained traditional DLs with the proposed model using SFD3. To evaluate the performance of our model, we used the Stochastic Gradient Descent (SGD) optimizer with 50 epochs. The training and validation accuracy are illustrated in Figure 6a, while Figure 6b illustrates the training and validation loss, where the confusion matrix of our experimental results is provided in Figure 7. It is clearly shown in the graph that training and validation accuracy of the proposed model are significantly increasing with each epoch, while our proposed model converged above 90% approximately within a few numbers of epochs. After reaching 30 epochs, the model accuracy or loss line graph did not change further and continues as a straight line, until the training process ends.

Furthermore, the proposed model is compared in terms of evaluation metrics with Stacking Ensemble [2], ConvoNet [13], HRRN [17], VGG19 [20] without pretrained weights, and Drive-Net [21]. We notice that Stacking Ensemble obtained 97.00% accuracy using SFD3 dataset as provided in Table 4.

**Figure 8:**
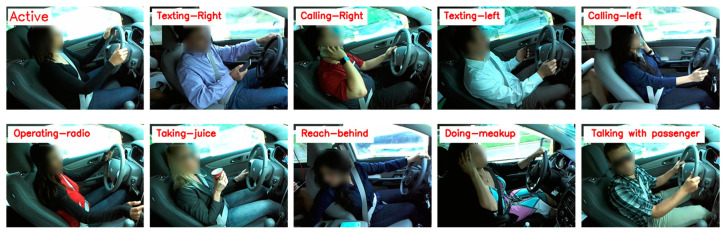
Visualized result of proposed model in real-time scene using SFD3 dataset.

In addition, ConvoNet achieved 98.48% performance in terms of accuracy, while the HRRN method has 96.23% accuracy based on the SFD3 dataset. Comparably, VGG19 and Drive-Net obtained 99.39% and 95% performance in terms of accuracy, respectively, the details are listed in Table 4. Our proposed model surpasses these methods by achieving higher accuracy, which is 99.58% accuracy using SFD3 dataset. In addition, the visual results of our proposed model using SFD dataset are shown in Figure 8.

### 4.5. Results Evaluation Using AUCD2 Dataset

**Figure 9:**
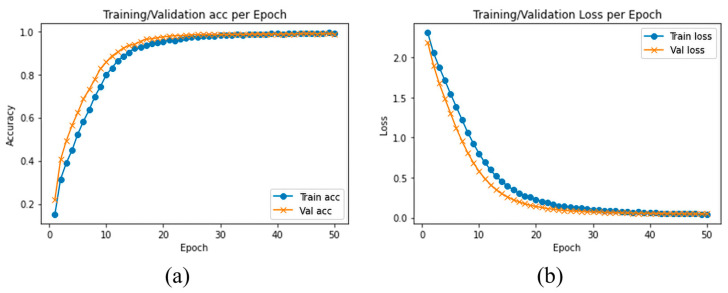
(**a**) Training accuracy and validation accuracy, (**b**) training loss and validation loss using AUCD3 dataset.

**Figure 10:**
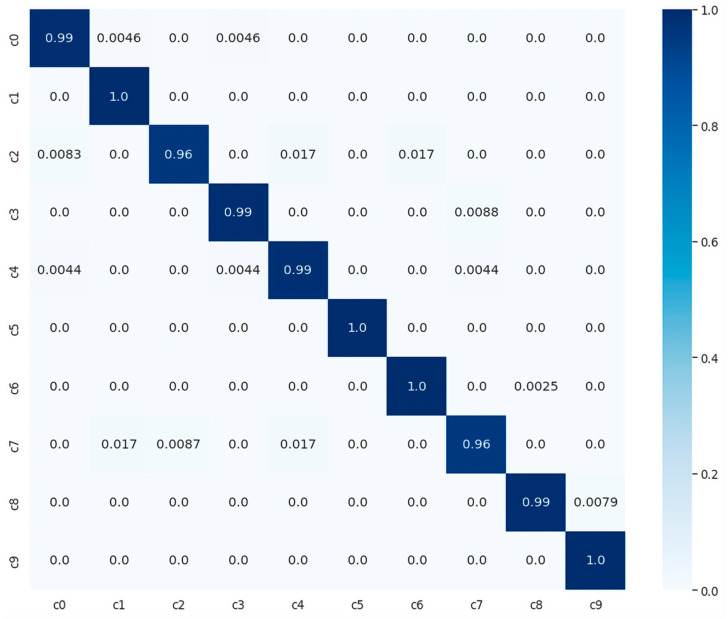
Confusion matrix of proposed model with normalized prediction between zero and one, using AUCD2 dataset.

Detailed reports of each model across test data using AUCD2 are presented in Table 5. We trained several baseline models for 50 epochs where the proposed model achieved optimal results compared with other models in terms of testing accuracy and testing loss as we observe in Table 5. The training and validation graphs of the proposed method using AUCD2 dataset are shown in Figure 9. Furthermore, the classification reports of the proposed model can be retrieved from the confusion matrix as presented in Figure 10.

**Figure 11:**
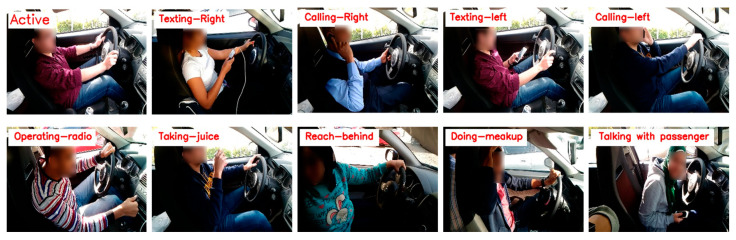
Visualized result of proposed model in real-time scene using AUC2 dataset.

We compared the proposed model using AUCD2 dataset with HRRN [17], C-SLSTM [22], D-HCNN [15], and ConvNet [13], where we examine that HRRN [17] obtained 92.36% accuracy using AUCD2 dataset. In addition, C-SLSTM [22] achieved 92.70% performance in terms of accuracy. Similarly, the D-HCNN [15] and ConvNet [13] methods have 95.59% and 95.64% accuracy, respectively. The proposed model obtained the highest accuracy, 98.97%, using the AUCD2 dataset as mentioned in Table 5. Moreover, Figure 11 is the visual results of the proposed model.

### 4.6. Ablation Study

This section provides the discussion and results over several DL-based models with and without CA mechanism. The comparison of proposed model with other DL-based models using evaluation metrics such as, F1-score, precision, recall, testing accuracy, and testing loss over SFD3 and AUCD2 datasets are briefly explained in the following subsequent sections.

The proposed model and other baselines were trained for 50 epochs with 32 batch size using a low learning rate of 0.001. Further, we set Stochastic Gradient Descent (SGD) with a momentum of 0.9 to ensure that the network retains most of the previously learned information. In these experiments, the proposed model was used to update the learning parameters moderately, which resulted in optimal performance on the target dataset. Additionally, we used the default input size (224 × 224) for each network.

In the experimental results, we conducted extensive experiments to evaluate the effectiveness of the proposed model and other baselines with and without CA for driver distraction detection using SFD3 and AUC2. We compared these models using several evaluation metrics including F1-score, precision, recall, testing accuracy, and testing loss. Our experimental results indicate that the models with CA outperforms among the models without CA across all metrics, indicating that the inclusion of CA enhances the proposed model’s effectiveness. 

Significantly, the proposed model with CA achieved an F1-score of 1.00, precision of 1.00, recall of 1.00, testing accuracy of 0.9958, and testing loss of 0.0202 for the SFD3 dataset as provided in Table 6. Furthermore, the proposed model with CA also obtained promising performance using the AUC2 dataset based on F1-score, precision, recall, testing accuracy, and testing loss, which were 0.99, 0.99, 0.99, 0.9897, and 0.0425, respectively as tabulated in Table 7. These results justify that the CA can help the model better attend to important features in the input data, which lead to enhancing the model performance.

#### 4.6.1. Ablation Study over SFD3 Dataset 

The classification reports of all the models across test data using SFD3 are presented in Table 6 where the proposed model achieved 0.9958 testing accuracy and 0.0202 testing loss. We observe that the proposed model is comparatively better, which exhibits the efficiency of our model. 

#### 4.6.2. Ablation Study over AUC2 Dataset 

Table 7 shows the results over AUCD2 dataset, where the VGG16 and VGG16+CA obtained the worst results in the experiments. Similarly, ResNet50 and ResNet50+CA also achieved the lowest results comparatively, which is approximately the same as shown in the Table 7. To compare with Xception and Xception+CA, these models achieved optimal performance in terms of testing accuracy. However, it is not suitable to deploy on resource constraint devices. Furthermore, InceptoinV3 and InceptionV3+CA achieved better results; however, the proposed model achieved the highest performance based on testing accuracy. In addition, we proposed this model due to the highest performance and lightweight model capabilities. These two reasons prove that it can be easily deployed on resource constraint devices.

### 4.7. Time Complexity

In the visual domain, achieving lower time complexity is a more challenging task than obtaining promising performance and achieving the smallest error rate in real time. Therefore, we compared the proposed model with four different baseline methods in terms of inference time. In addition, numerous experiments are conducted based on two different hardware such as CPU and GPU as tabulated in Table 8. In these experiments, the ResNet50 and ResNet50+CA have lower inference speed than the InceptioV3 and InceptioV3-CA. The proposed model achieved higher frame per second (FPS) rates for both CPU and GPU than other baseline models, which is 21.73, and 83.75, respectively. In addition, the inference time of the proposed EFFNet-CA can be further enhanced based on hardware improvement. Hence, the inference speed justifies that the proposed model can be easily deployed over resource constraints for real-time decision-making.

## 5. Conclusions

Driver distraction leads drivers toward accidents that affect lives, i.e., driver death or major injuries and causes of economic losses, globally. In the literature, several techniques have been introduced to detect driver distraction in an efficient way. However, their techniques are time-consuming, have a high false alarm rate and are difficult to deploy on edge devices due to the high number of parameters. To solve a certain problem, we proposed a novel framework for an efficient and effective driver distraction based on a CNNs with the integration of CAmechanism. Moreover, the proposed model contains three steps, such as training, testing and evaluation. Additionally, our proposed model is compared with various baseline CNNs where only the classification layers were fine-tuned while the rest of the models’ layers were frozen. Moreover, the proposed model achieved optimal results in terms of testing accuracy and testing loss using two well-known datasets. The proposed model indicated 99.58% testing accuracy using the SFD3 dataset and 98.97% testing accuracy on the AUCD2 dataset. In other words, the proposed model can easily be deployed on resource constraints devices due to its size and less computational complexity. Further, due to the rapid increase in the developing technologies, the metaverse provides us a great opportunity for better contributions such as the implementation of our proposed work in metaverse-based 3D modeling.

In the future, our goal is to make the proposed model more effective, reduce the false alarm rate, and try to reduce the number of parameters of using model compression techniques such as pruning and quantization. Furthermore, we also aim to deploy our proposed architecture on resource constraints such as Raspberry Pi and Jetson Nano.

## Figures and Tables

**Table 1:** Summaries of related articles.

Reference	Description	Method
[12]	Proposed an ensemble-based technique for the classification of driver distraction.	DL Ensemble Technique
[13]	Utilized a deep learning architecture based on CNN for driver distraction detection using two well-known datasets.	DL
[14]	Implemented two DCNN pretrained networks named (InceptionV3 and Xception) for the recognition of driver action using publicly available SFD3 dataset.	DCNN
[3]	The authors implemented several architectures namely, vanilla CNN with and without augmentation technique, and pretrained CNN model for driver distraction detection.	Vanilla CNN
[1]	The authors implemented a 3D CNN technique for driver behavior monitoring.	3D CNN
[15]	Utilized a novel D-HCNN algorithm, which detects driver action in early stages while driving using AUC2 and SFD3 datasets.	D-HCNN
[16]	Proposed an ensemble technique which contains four DL pretrained architectures using video data.	DL Ensemble Technique
[17]	Trained a DL pipeline named inception using some fine-tunning strategies for accurate classification of driver behaviors.	DL
[2]	Used a stacking technique for obtaining optimal results. Initially, they stacked all the feature vectors and feed to the CNN for training purposes.	Stacking Ensemble Technique
[19]	The authors presented a deep learning framework called DarNet which classifies driver behavior using input sensor data.	DL
[20]	The researchers utilized the deep convolutional neural network for efficient and effective classification of driver distraction using the SFD3 dataset. In addition, their experimental results are focused on three rounds of 5-fold cross validation.	DCNN
[21]	The authors used forward machine learning based on convolution neural network which not only classifies the driver’s distraction but also finds the reason of their distraction.	ML and CNN
[22]	Presented a method named Drive-Net based on supervised learning for the accurate detection of driver behavior while driving using the well-known publicly available SFD3 dataset.	DL
[23]	The authors introduced a novel framework called LSTM to detect online driver activity.	DL LSTM
[24]	Evaluated three CNN-based transfer learning techniques using some fine-tuning strategies for the recognition of seven common driver distractions using low-cost camera collected images.	CNN

**Table 2:** Briefly detail of SFD3 dataset.

Class	Class Name	Number of Images
c0	Safe driving	2489
c1	Texting-right	2267
c2	Calling on the phone—right	2317
c3	Texting—left	2346
c4	Calling on the phone—left	2326
c5	Operating the Radio	2312
c6	Drinking	2325
c7	Reaching behind	2002
c8	Makeup	1911
c9	Talking to the passenger	2129
Total	---	22,424

**Table 3:** Brief detail of AUCD2 dataset.

Class	Class Name	Number of Images
c0	Safe driving	2706
c1	Texting-right	1438
c2	Calling on the phone—right	976
c3	Texting—left	844
c4	Calling on the phone—left	1040
c5	Operating the Radio	843
c6	Drinking	796
c7	Reaching behind	754
c8	Makeup	764
c9	Talking to the passenger	1517
Total	---	11,678

**Table 4:** Comparison between proposed model with different SOTA model using SFD3 dataset.

Reference	Accuracy
Stacking Ensemble [2]	97.00%
ConvoNet [13]	98.48%
HRRN [17]	96.23%
VGG19 without pretrained weight [21]	99.39%
Drive-Net [22]	95.00%
**Proposed Model**	**99.58%**

**Table 5:** Comparison between proposed model with different SOTA models using AUCD2 dataset.

Reference	Accuracy
HRRN [17]	92.36%
C-SLSTM [41]	92.70%
D-HCNN [15]	95.59%
ConvNet [13]	95.64%
**Proposed Model**	**98.97%**

**Table 6:** Classification reports of different pretrained models using SFD3 dataset.

Model	F1-Score	Precision	Recall	Testing Accuracy	Testing Loss
VGG16	0.88	0.88	0.87	0.8792	0.4214
VGG16+CA	0.93	0.93	0.93	0.9332	0.2453
ResNet50	0.94	0.94	0.94	0.9394	0.5736
ResNet50+CA	0.98	0.98	0.98	0.9804	0.1201
Xception	0.96	0.96	0.96	0.9611	0.1929
Xception+CA	0.97	0.97	0.97	0.9671	0.1351
InceptionV3	0.87	0.89	0.87	0.8810	0.8923
InceptionV3+CA	0.91	0.92	0.91	0.9178	0.5596
EfficientNetB0	0.98	0.98	0.97	0.9760	0.0961
**Proposed Model**	**1.00**	**1.00**	**1.00**	**0.9958**	**0.0202**

**Table 7:** Classification reports of different pretrained models using AUCD2 dataset.

Model	F1-Score	Precision	Recall	Testing Accuracy	Testing Loss
VGG16	0.92	0.93	0.91	0.9282	0.2778
VGG16+CA	0.96	0.96	0.95	0.9618	0.1634
ResNet50	0.95	0.95	0.94	0.9562	0.2809
ResNet50+CA	0.95	0.96	0.95	0.9582	0.3028
Xception	0.96	0.96	0.96	0.9681	0.2145
Xception+CA	0.97	0.97	0.98	0.9767	0.1035
InceptionV3	0.95	0.96	0.94	0.9533	0.3039
InceptionV3+CA	0.94	0.95	0.93	0.9510	0.2592
EfficientNetB0	0.99	0.99	0.99	0.9880	0.0598
**Proposed Model**	**0.99**	**0.99**	**0.99**	**0.9897**	**0.0425**

**Table 8:** Time complexity between proposed model and other baseline models for CPU and GPU.

Reference	Frame per Second		Parameters (Million)	Model Size (MB)
	CPU	GPU		
ResNet50	8.37	57.3	23.58	98
ResNet50+CA	6.00	53.45	24.37	99
InceptionV3	12.90	70.55	21.80	92
InceptionV3-CA	10.55	66.10	22.59	94
Proposed Model	21.73	83.75	4.57	5
